# Supplementary material for: The interactome of intact mitochondria by cross-linking mass spectrometry provides evidence for coexisting respiratory supercomplexes
Source: Mol Cell Proteomics. 2017 Dec 8;17(2):216–32. doi: 10.1074/mcp.RA117.000470 (PMC5795388; doi:10.1074/mcp.RA117.000470)
Supplement: Supplemental Data [file supp_RA117.000470_133922_0_supp_23978_fzffwf.zip › spectra_annotation/mito_spectra_annotation/232-1-10-1-3-1.pdf]

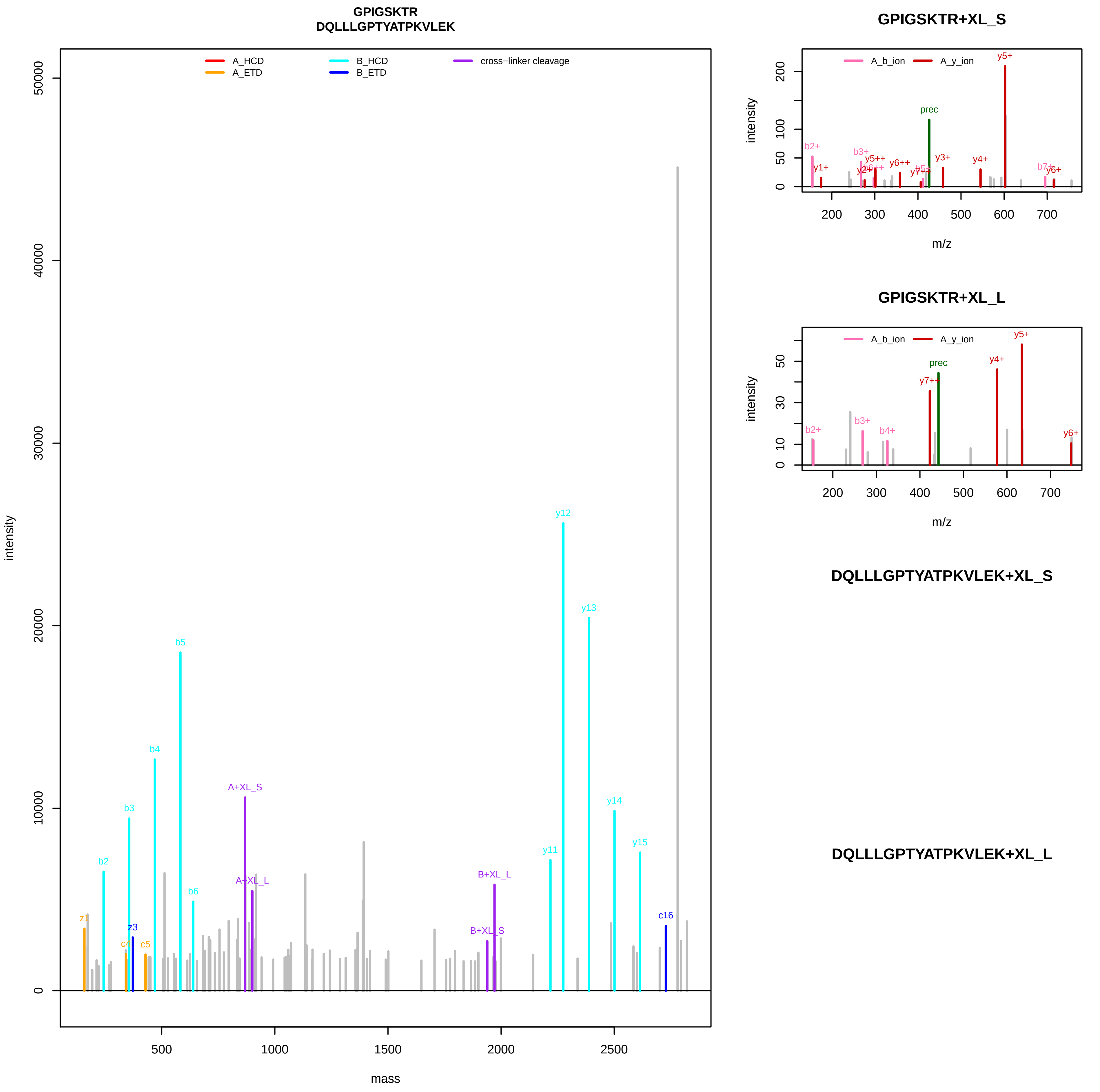
GPIGSKTR
DQLLLGPTYATPKVLEK
A_HCD
A_ETD
B_HCD
B_ETD
cross−linker cleavage
intensity
mass
0
10000
20000
30000
40000
50000
500
1000
1500
2000
2500
z1
b2
b3
c4
z3
c5
b4
b5
b6
A+XL_S
A+XL_L
B+XL_S
B+XL_L
y11
y12
y13
y14
y15
c16
GPIGSKTR+XL_S
A_b_ion
A_y_ion
intensity
m/z
prec
b2+
y1+
b3+
y2+
y5++
y6++
y7++
b5+
y3+
y4+
y5+
b7+
y6+
GPIGSKTR+XL_L
A_b_ion
A_y_ion
intensity
m/z
prec
b2+
b3+
b4+
y7++
y4+
y5+
y6+
DQLLLGPTYATPKVLEK+XL_S
DQLLLGPTYATPKVLEK+XL_L
